# Bone health in myelodysplastic neoplasms: results from the prospective longitudinal BoHemE study

**DOI:** 10.1038/s41375-026-03125-8

**Published:** 2026-10-09

**Authors:** Heike Weidner, Elena Tsourdi, Anne Sophie Platzbecker, Martina Rauner, Ulrike Baschant, Maria G. Ledesma-Colunga, Karolina Basiak, Katja Sockel, Ekaterina Balaian, Susann Winter, Anna Harig, Klaus H. Metzeler, Katharina S. Götze, Dominic Brauer, Nicole Modler, Michael Cross, Marie Schneider, Jörg Lützner, Andreas Roth, Thomas Zerjatke, Gabriele Müller, Uwe Platzbecker, Lorenz C. Hofbauer

**Affiliations:** 1 Division of Endocrinology, Diabetes, and Bone Diseases, Department of Medicine III & Center for Healthy Aging, Medical Faculty and University Hospital Carl Gustav Carus, Dresden University of Technology, Dresden, Germany; 2 Else Kroener Fresenius Center for Digital Health, Faculty of Medicine and University Hospital Carl Gustav Carus, Dresden University of Technology, Dresden, Germany; 3 Medizinische Universität Lausitz – Carl Thiem, Cottbus, Germany; 4 Digital Health Cluster, Department for Digital Health, Economics & Policy, Hasso-Plattner Institute, University of Potsdam, Potsdam, Germany; 5 Department of Medicine I, Medical Faculty and University Hospital Carl Gustav Carus, Dresden University of Technology, Dresden, Germany; 6 German Cancer Consortium (DKTK), Partner Sites Dresden and Munich and German Cancer Research Center (DKFZ), Heidelberg, Germany; 7 Department of Hematology, Cell Therapy, Hemostaseology, and Infectious Diseases, University Hospital, Leipzig, Germany; 8 Department of Medicine III, Technical University of Munich, School of Medicine and Health, Munich, Germany; 9 Department of Orthopedic Surgery, University Hospital Carl Gustav Carus, Dresden University of Technology, Dresden, Germany; 10 Division of Orthopedic, Trauma and Plastic Surgery, University Hospital, Leipzig, Germany; 11 Faculty of Medicine Carl Gustav Carus, Institute for Medical Informatics and Biometry, Dresden University of Technology, Dresden, Germany; 12 Center for Evidence-Based Healthcare, Medical Faculty and University Hospital Carl Gustav Carus, University Hospital Carl Gustav Carus, Dresden University of Technology, Dresden, Germany

**Keywords:** Translational research, Biostatistics

## Abstract

Myelodysplastic neoplasms (MDS) are characterized by insufficient hematopoiesis and alterations of the bone marrow microenvironment. However, bone health in MDS has not been evaluated in a prospective, longitudinal setting. Within the **Bo**ne and **Hem**atology in **E**lderly (BoHemE) study, bone mineral density (represented as T-score) and bone-specific serum markers were assessed in patients with MDS (*n* = 111; 36% female) and age-matched controls (*n* = 84; 55% female) at baseline and after 3 years. At baseline, patients with MDS had a lower T-score [−0.2; *p* < 0.05] compared to controls, which was mainly attributed to the lower T-score in women with MDS [−0.3; *p* < 0.05]. Furthermore, the *DNMT3A* mutation was associated with a lower T-score within the MDS cohort [−0.7; *p* < 0.05], but the occurrence in controls had no effect. Albumin-adjusted calcium and phosphate levels correlated with T-scores within the MDS cohort [*p* < 0.0001 and *p* < 0.01, respectively], independent of age, sex, MDS duration, IPSS-M score, or transfusion dependence. Quality of life was lower in patients with MDS [*p* < 0.01], and remained stable over 3 years, whereas T-scores declined [−0.3; *p* < 0.05]. The presence of osteoporosis was associated with poor overall survival in MDS [*p* < 0.01]. In conclusion, bone health should be evaluated as part of the diagnostic work-up and clinical management of MDS.

## Introduction

The osteo-hematopoietic niche constitutes a highly dynamic microenvironment in which hematopoietic stem and progenitor cells (HSPCs) closely interact with mesenchymal stromal cells, osteoblasts, osteoclasts, endothelial cells, and immune components. Balanced, bidirectional interactions within this niche are essential for maintaining effective hematopoiesis and bone homeostasis: stromal signals regulate HSPC fate, while HSPC reciprocally shape bone remodeling [1]. Disruption of this finely tuned niche has been described in myelodysplastic neoplasms (MDS) [2].

MDS comprise a heterogeneous group of stem cell disorders and represent one of the most common hematologic neoplasms in the elderly. Typical features include ineffective hematopoiesis, peripheral cytopenias, and an increased risk of progression to acute myeloid leukemia. Beyond clonal hematopoiesis (CH), dysregulation of the bone marrow microenvironment contributes to disease initiation and progression [3–5]. Thus, the niche undergoes profound remodeling, evolving from a pro-inflammatory state to an immunosuppressive environment in advanced MDS, thereby promoting CH and hematopoietic insufficiency [6, 7].

Given the intimate coupling between hematopoiesis and bone metabolism [8], impaired HSPC function in MDS may also affect bone remodeling. Osteoporosis is a common condition in the elderly and dual-energy X-ray absorptiometry (DXA) represents the clinical standard to assess bone mineral density (BMD). The prevalence of osteoporosis increases continuously after the age of 50 and is higher in women. In individuals aged 70–79 years, the prevalence increases to 4% in men and 20% in women [9]. In men, osteoporosis is linked to reduced quality of life (QoL) due to pain, immobility, and reduced social activities [10]. In addition to aging, chronic inflammation, comorbidities, and certain medications further promote bone loss and increase the fracture risk in both sexes.

In a retrospective insurance-based study of >890,000 subjects, we reported that women and men with MDS had a 2- to 3-fold higher osteoporosis prevalence compared to age-matched individuals without MDS [11]. However, bone health has not yet been comprehensively assessed in MDS. Here, we determined bone health in patients with and without MDS in the prospective, longitudinal Bone and Hematology in Elderly (BoHemE) trial.

## Methods

### Patient and control cohorts

The BoHemE study (July 2017 to August 2024, NCT02867085) evaluated the interactions between bone metabolism and hematopoiesis in patients with MDS and elderly individuals without known hematologic disease. The study was conducted at the University Hospital Dresden and the University Hospital Leipzig, Germany after approval by local Ethics Committees (Dresden: EK 393092016; Leipzig: 137/19-lk) and conducted in accordance with the Declaration of Helsinki and the International Conference on Harmonization for Good Clinical Practice. Written informed consent was obtained from all participants.

The BoHemE study included patients older than 60 years with MDS and individuals without hematological disease undergoing total hip replacement surgery for osteoarthritis as a control group. Exclusion criteria were renal insufficiency (estimated glomerular filtration rate (eGFR) < 45 ml/min/1.73 m^2^), liver cirrhosis, active infection, heart failure, and dementia (Supplementary Table S1). Patients taking osteoporosis medications other than vitamin D and calcium were excluded. At baseline, demographic and physical characteristics were documented, and the body mass index (BMI) was calculated. Medical history, comorbidities, known falls and fractures were documented. All participants underwent DXA scan, and blood samples were collected. QoL was evaluated using the EORTC QoL Questionnaire (QLQ-C30, version 3). During follow-up, controls attended the outpatient clinic every six months over a 3-year period, with blood analyses at each visit. Samples from patients with MDS were collected during this interval. Updated information on physical characteristics, medical history, and QoL was obtained annually from all participants. BMD measurements were repeated after 3 years.

The primary endpoint of the BoHemE study was to assess BMD at the lumbar spine, total hip, and femoral neck in patients with MDS and the control group. Secondary endpoints were the impact of age, sex, molecular International Prognostic Scoring System (IPSS-M) risk score, molecular genetics, iron parameters, MDS duration, QoL, MDS evolution over 3 years, and overall survival on bone health in MDS.

### DXA measurement

BMD was measured by DXA at the lumbar spine (L1–L4), total hip, and femoral neck at baseline and after three years, using GE DPX Prodigy (until April 2021), and GE Lunar iDXA (from May 2021 onward, both GE HealthCare, Chicago, Illinois, USA) in Dresden, and Hologic Horizon A in Leipzig (Hologic, Berlin, Germany). BMD values were transformed into T-scores, and the lowest T-score across all sites was used to diagnose osteoporosis (<−2.5), osteopenia (between −1 and −2.5), or a normal BMD (> −1) [12]. Due to degenerative alterations at the spine, valid DXA scans could not be evaluated in all individuals. In addition, to minimize the bias of increased BMD at the total hip associated with hip osteoarthritis, T-scores of the femoral neck are presented in the results only.

### Serum/plasma parameters

Blood samples were obtained at baseline and follow-up visits. Hematologic parameters were analyzed using XN-1000 (Dresden) and XN-9000 (Leipzig) (Sysmex, Hamburg, Germany). All other samples for plasma collection were centrifuged immediately and stored at -80 °C until centralized analysis in Dresden. These included calcium, phosphate, and creatinine (Roche Diagnostics, Mannheim, Germany), and 25-hydroxy vitamin D (25-OH D3) (IDS Immunodiagnostic Systems, Frankfurt/Main, Germany), assessed by ELISA. Calcium levels were adjusted for albumin. Bone-specific alkaline phosphatase (BSAP) (IDS Immunodiagnostic Systems) and parathyroid hormone (PTH) (Roche Diagnostics) were analyzed, while cross-linked C-telopeptide of type I collagen (CTX-I) and osteocalcin were measured by ELISA (both IDS Immunodiagnostic Systems).

### EORTC QoL questionnaires

The EORTC QLQ-C30 (version 3.0) is a 30-item questionnaire comprising five functional scales (physical, role, emotional, cognitive, and social), three symptom scales (fatigue, nausea/vomiting, and pain), six single items (dyspnea, insomnia, appetite loss, constipation, diarrhea, and financial difficulties), and a global health status/QoL scale to determine health-related QoL in patients with malignant disease [13]. All items are scored on a four-point response scale from 1 (not at all), 2 (a little), 3 (quite a bit) to 4 (very much) except for the overall health and overall QoL, where the scale ranges from 1 (very bad) to 7 (excellent). Raw scores for each scale/item were linearly transformed to a 0–100 scale according to the EORTC QLQ-C30 scoring manual (3rd edition, 2001, Brussels). Missing items were handled by imputation according to the recommended guidelines.

### Statistical analysis

Statistical analyses were performed using R and in GraphPad Prism V10.5.0. General characteristics and calculated scores for QoL of the cohorts are presented as medians with the interquartile range (IQR) in tables. Numeric data were depicted as violin plots, and the distribution for T-score per cohort was visualized in percentages.

Differences between the two groups were assessed using the Mann–Whitney U tests, and Chi-squared tests were used to analyze the distribution of T-scores within the two groups. Linear regression was applied to assess the association between an outcome and an independent variable, while multiple linear regression was used to evaluate the effects of several independent variables simultaneously. To fit model assumptions, osteocalcin, CTX-I, BSAP, and ferritin levels, as well as the MDS duration were log-transformed prior to inclusion in the regression models. Regression estimates (β coefficients) and p-values are reported in the tables.

The Wilcoxon signed-rank test was used to compare baseline and follow-up measurements within the same patient. Kaplan–Meier analyses and log-rank tests for trend were performed to compare overall survival across T-score groups. Statistical significance was defined as *p*-value < 0.05.

## Results

### Baseline characteristics of the study population

**Fig. 1:**
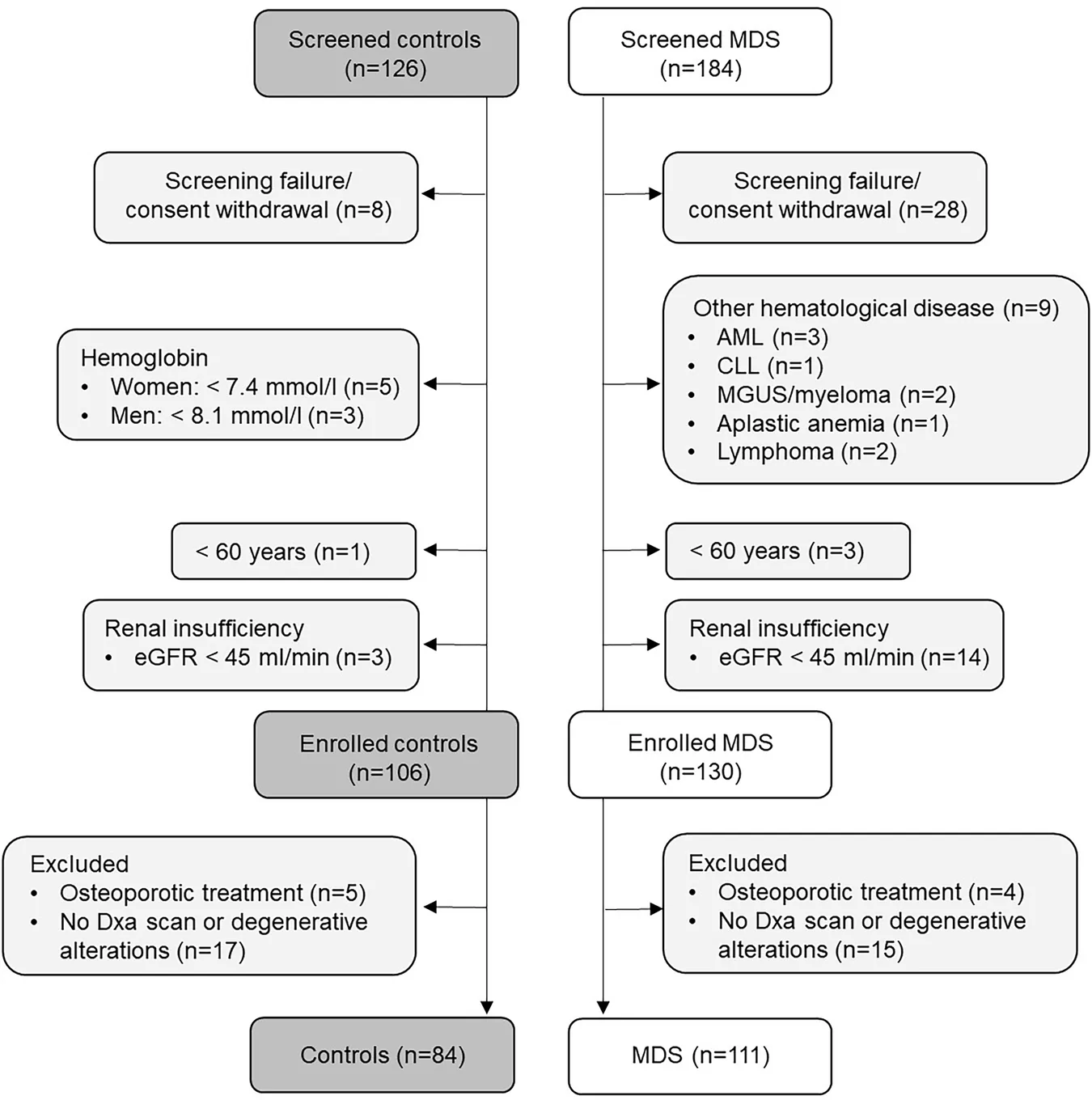
Flowchart of selection criteria for the BoHemE study. The flowchart illustrates the study selection process, including all exclusion criteria from screening and enrollment to the final number of participants.

Overall, 310 subjects were screened (184 patients with MDS, 126 controls). After exclusion based on predefined criteria (bone-specific therapy; non-assessable femoral BMD by DXA), 111 patients with MDS and 84 controls were included in the final analysis (Fig. 1). The median age was similar across both cohorts. The MDS cohort included 40 women (median age: 68 years) and 71 men (median age: 69 years), while the control cohort included 46 women (median age: 69 years) and 38 men (median age: 68 years). BMI was comparable between women with or without MDS, but significantly lower in men with MDS compared to male controls [−8%; *p* < 0.0001] (Table 1). The median duration since MDS diagnosis was 13 months. According to WHO classification, most patients were diagnosed with MDS with increased blasts (40%), MDS with low blasts (24%), and MDS with low blasts and *SF3B1* mutation (13%). The cohort also included 13 patients with chronic myelomonocytic leukemia (CMML). As CMML was not an exclusion criterion, these patients were included and considered separately only for hematological analysis. Based on IPSS-M, disease risk was similarly distributed between low-risk (54%) and high-risk (46%) groups, with very low, low, and moderate low being combined, as were moderate high, high, and very high (Table 2). During screening, all patients were asked whether they had sustained any falls or fractures regardless of whether a temporal association existed between the two events. Interestingly, 23% of the controls reported a fall and 19% a previous fracture (vertebral, non-vertebral/hip, or radius fracture), whereas only 6% of the patients with MDS [*p* < 0.001] reported a fall and 6% a fracture [*p* < 0.05]. The proportion of patients receiving vitamin D supplementation for more than 3 months was similar in both cohorts (Table 1).

**Table 1:** Characteristics of BoHemE study participants.

	Controls (*n* = 84)	MDS cohort (*n* = 111)
Sex, *n*		
Women	46 (55%)	40 (36%)
Men	38 (45%)	71 (64%)
Age, years		
All	68 (63–75)	68 (65–73)
Women	69 (63–76)	68 (64–73)
Men	68 (64–73)	69 (65–73)
BMI, [kg/m^2^]		
All	27.8 (25.6–32.4)	26.6 (24.2–28.2)**
Women	26.6 (23.9–28.1)	25.8 (23.6–27.6)
Men	29.4 (27.8–34.2)	27.0 (24.6–29.4)****
T-score		
All	−1.0 (−1.8 to −0.1)	−1.2 (−2.0 to −0.5)*
Women	−1.3 (−1.8 to −0.2)	−1.6 (−2.1 to −0.8)*
Men	−0.8 (−1.5 to 0.1)	−1.0 (−1.9 to −0.4)
Falls are known, *n*		
Yes	19 (23%)	5 (6%)***
No	64 (77%)	85 (94%)
No information	1	21
Fractures are known, *n*		
Yes	16 (19%)	6 (6%)*
Vertebral	5	3
Non-vertebral/hip	11	3
Radius fracture	4	–
No	67 (81%)	87 (94%)
No information	1	18
Vitamin D supplementation at the time of screening (>3 months)		
Yes	9 (11%)	14 (13%)
No	75 (89%)	97 (87%)
Diseases and comorbidities, *n*		
Cancer^a^	8 (10%)	17 (15%)
Chronic kidney disease	1 (1%)	5 (5%)
Diabetes mellitus	12 (14%)	18 (16%)
Rheumatoid arthritis	2 (2%)	3 (3%)
Thyroid diseases	8 (10%)	21 (19%)

For continuous variables median (1st–3rd quartile) are given.

*p < 0.05, **p < 0.01; ***p < 0.001, ****p < 0.0001 vs. controls.

^a^Except blood cancer.

**Table 2:** Classification of the MDS cohort.

	MDS cohort (*n* = 111)
Time since MDS diagnosis, [months]	
All	13 (4-37)
Women	11 (4-29)
Men	18 (5-40)
WHO 2022 classification, *n*	
MDS-5q	5 (4%)
MDS-SF3B1	14 (13%)
MDS-biTP53	2 (2%)
MDS-LB	27 (24%)
MDS-LB and RS	3 (3%)
MDS-h	1 (1%)
MDS-IB	44 (40%)
MDS-f	-
CMML	15 (13%)
IPSS-M risk group, *n*^a^	
Very low	5 (5%)
Low	30 (31%)
Moderate low	17 (18%)
Moderate high	10 (11%)
High	19 (20%)
Very high	14 (15%)
Transfusion-dependent, *n*	
Yes	33 (30%)
No	78 (70%)

*WHO* World Health Organization, *MDS-5q* MDS with low blasts and isolated 5q deletion, *MDS-SF3B1* MDS with low blasts and SF3B1 mutation, *MDS-biTP53* MDS with biallelic TP53 inactivation, *MDS-LB and RS* MDS with low blasts and ring sideroblast, *MDS-LB* MDS with low blasts, *MDS-IB* MDS with increased blasts-1/-2, *MDS-h* hypoplastic MDS, *MDS-f* MDS with fibrosis, *CMML* chronic myelomonocytic leukemia-0/-1/-2, *IPSS-M* International Prognostic Scoring System-molecular.

^a^Without patients with CMML and one patient with MDS with VEXAS syndrome.

### Primary endpoint - MDS is associated with lower T-score

**Fig. 2:**
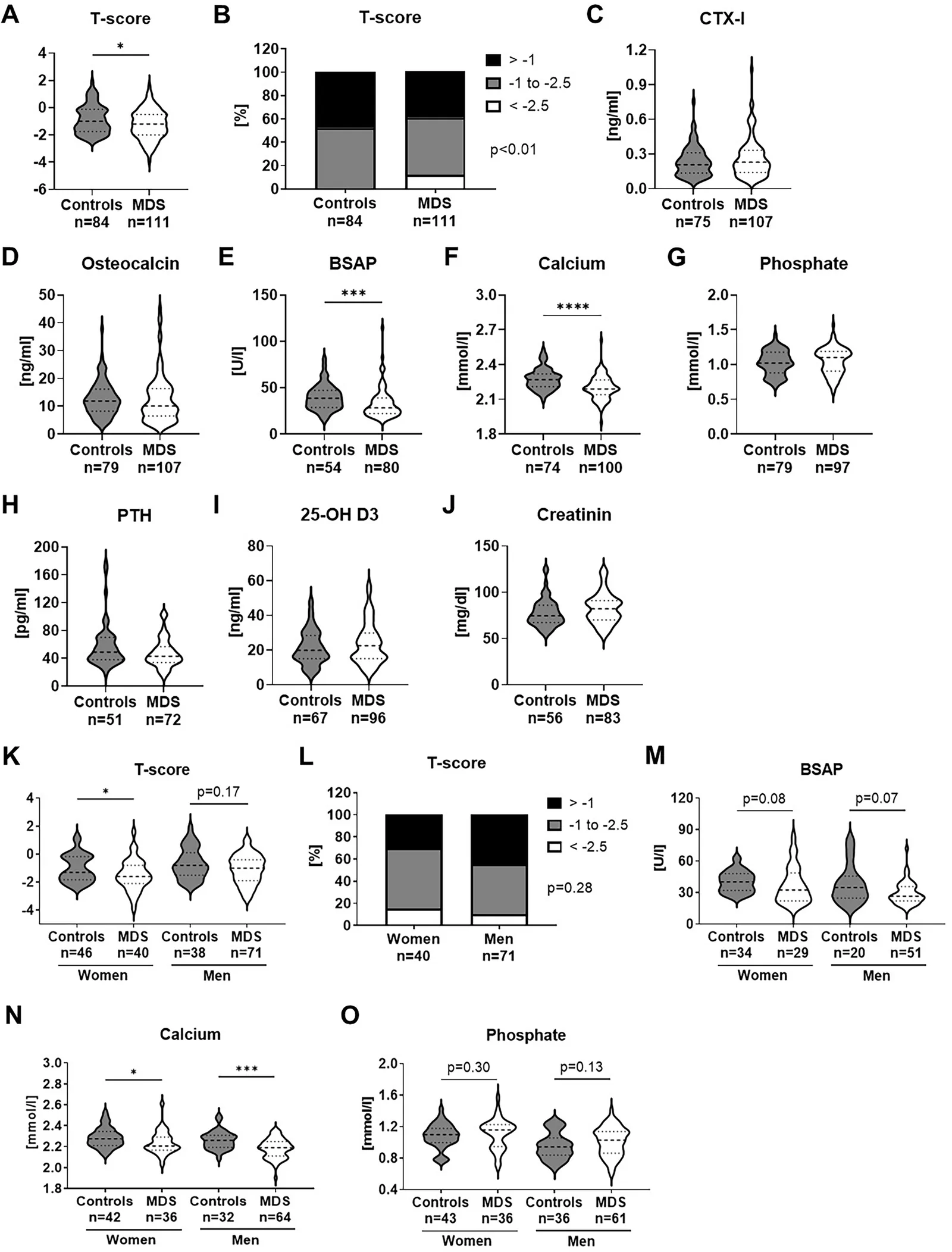
Low femoral neck T-score in MDS is associated with reduced calcium. DXA scans were performed in all participants at baseline. Bone mineral density was transformed into T-scores (**A**), and subjects were classified as normal (T-score > −1.0), osteopenia (T-score between −1.0 and −2.5), and osteoporosis (T-score < −2.5) according to WHO classification. **B** Comparison of the femoral neck T-score distribution in controls and patients with MDS. **C** Cross linked C-telopeptide of type I collagen (CTX-I) and **D** osteocalcin were determined in plasma samples. **E** Bone-specific alkaline phosphatase (BSAP), **F** albumin-adjusted calcium, **G** phosphate, **H** parathyroid hormone (PTH), **I** 25-hydroxy vitamin D3 (25-OH D3), and **J** creatinine levels were measured in serum using ELISA. Comparison of **K** the femoral neck T-score and **L** its distribution, **M** BSAP, **N** albumin-adjusted calcium, and **O** phosphate in women and men between controls and patients with MDS. **B**, **L** Data are shown as percentage distribution. Statistical analysis was performed by Chi-squared test. All other data are shown as median with interquartile range in violin plots. Women and men were analyzed separately. The Mann–Whitney U test was used for the comparison of two groups. *p < 0.05; **p < 0.01; ***p < 0.001; ****p < 0.0001.

As expected, patients with MDS and CMML had significantly reduced red blood cell (RBC) and platelets counts, whereas the leukocyte counts were only reduced in MDS compared to controls [*p* < 0.0001] (Supplementary Fig. S1A–C). The median femoral neck T-score was lower in the MDS cohort compared to controls [−0.2; *p* < 0.05] due to a higher proportion of osteoporotic T-scores [12 vs. 0; *p* < 0.01] (Fig. 2A, B).

To explore potential mechanisms underlying the osteoporotic phenotype in MDS, biochemical markers of bone turnover were analyzed, since patients with osteoporosis frequently display increased bone turnover [14]. However, the resorption marker CTX-I and the formation marker osteocalcin were comparable between patients with MDS and controls, whereas the mineralization marker BSAP was reduced in patients with MDS [−27%, *p* < 0.001] (Fig. 2C–E). Patients with MDS had lower serum albumin-adjusted calcium levels [−4%; *p* < 0.0001] than controls, however, median values remained within the normal range. Serum phosphate levels did not differ between groups (Fig. 2F, G). Median serum PTH and 25-OH vitamin D3 levels were similar between cohorts (Fig. 2H, I). The lower calcium levels were not attributable to impaired renal function, as all individuals with moderate to severe renal insufficiency were excluded and creatinine levels were comparable between groups (Fig. 2J).

In conclusion, MDS is associated with lower T-scores, calcium and BSAP serum levels.

Of note, the study cohort included 15 patients with CMML, a MDS/MPN overlap neoplasm. These patients had a median leukocyte count of 10 × 10⁹/L, indicating that the cohort predominantly comprised the myelodysplastic (MD-CMML) rather than the myeloproliferative subtype. Importantly, T-scores, bone mineral density categories, and all assessed bone parameters did not differ significantly between patients with CMML and those with other MDS subtypes (Supplementary Fig. S2A–F; Supplementary Table S2).

### Secondary endpoints

#### Decreased T-scores in women with MDS

To further investigate the decreased T-scores observed in MDS, the cohorts were stratified by sex. In women with MDS, the median T-score was −0.3 lower than in control women [*p* < 0.05]. No significant differences in T-scores were noted in men (Fig. 2K, Table 1). However, the distribution of the femoral neck T-scores showed a similar percentage in women compared to men with MDS (Fig. 2L). BSAP was lower in the overall MDS cohort compared to controls. However, this difference was not observed after stratification by sex. In contrast, both women and men with MDS exhibited lower albumin-adjusted calcium levels compared to sex-matched controls, whereas phosphate levels were similar between patients with MDS and controls (Fig. 2L–N).

### Lower T-scores are associated with high calcium and reduced phosphate in MDS

Using linear regression, we calculated the estimate whether the T-score (outcome) could be explained by bone-specific parameters. CTX-I and osteocalcin were negatively correlated with T-scores [β: −0.31 and −0.30, respectively; *p* < 0.05]. In contrast, BSAP had no effect on T-scores. The estimate indicated that the lowest serum calcium levels in patients with MDS were associated with higher T scores [β: −3.56; *p* < 0.001]. Conversely, phosphate showed a positive association with T-scores [β: 1.66; *p* < 0.01]. Other parameters, such as PTH and 25-OH D3, were not significantly associated with T-scores in patients with MDS (Table 3). Interestingly, none of the bone parameters had an influence on the T-score in controls (Supplementary Table S3).

**Table 3:** Association between the T-score and bone-specific parameters, age, sex, or MDS characteristics within the MDS cohort.

	Sample size, *n*	β	*p*-value
Linear regression with T-score as the outcome			
CTX-I (log)	107	−0.31	**<0.05**
Osteocalcin (log)	107	−0.30	**<0.05**
BSAP (log)	80	−0.42	0.15
Calcium	100	−3.56	**<0.001**
Phosphate	97	1.66	**<0.01**
PTH	72	0	0.82
25-OH D3	96	−0.01	0.46
Ferritin (log)	96	−0.24	0.24
Age	111	−0.02	0.28
Age			
60–69 years	62	ref.	0.35
≥ 70 years	49	−0.20	
Sex			
Women	40	ref.	
Men	71	0.42	0.05
Time interval since MDS diagnosis (log)	109	0.02	0.77
IPSS-M			
Low risk	52	ref.	
High risk	43	0.06	0.79
Transfusion dependency			
No	78	ref.	
Yes	33	0.12	0.59
Multiple regression with T-score (outcome)^a^			
Calcium	93	−3.56	**<0.001**
Phosphate	93	1.51	**<0.01**
Multiple regression with T-score (outcome) adjusted for calcium and phosphate^b^			
Age	93	−0.01	0.71
Age			
60–69 years	53	ref.	
≥ 70 years	40	−0.03	0.87
Sex			
Women	34	ref.	
Men	59	0.32	0.17
Time interval since MDS diagnosis (log)	93	−0.01	0.95
IPSS-M			
Low risk	45	ref.	
High risk	34	−0.10	0.66
Ferritin (log)	76	−0.06	0.57
Transfusion dependency			
No	65	ref.	
Yes	28	0.03	0.90
*DNMT3A* mutation			
No	93	ref.	
Yes		−0.46	0.09

*CTX-I* cross-linked C-telopeptide of type I collagen, *BSAP* bone-specific alkaline phosphatase, *PTH* parathyroid hormone, *25-OH D3* 25-hydroxyvitamin D3, *IPSS-M* International Prognostic Scoring System-molecular, *log* before use values were converted to logarithms, *ref* reference, *β* estimate.

Values in bold (<0.05, <0.01, and <0.001) indicate significant changes.

^a^R^2^ = 0.18 for this model.

^b^Multiple regression for T-score including calcium and phosphate plus each of the listed variables separately.

### Reduced T-score is not linked to age, sex, and transfusion dependency in MDS

Next, we assessed the association between the T-score (outcome) and age, sex, or MDS duration using linear regression. Neither age nor the stratification of the MDS cohort into two age groups (60–69 years and ≥70 years), nor sex, had an influence on T-scores (Table 3). In contrast, there was a negative association between age and the T-score in controls (Supplementary Table S3). Although MDS duration ranged from months to years (Table 2), it did not affect BMD (Table 3). In the MDS cohort, 30% of the patients had received RBC transfusion (≥2 RBC transfusion/month for more than 3 months) at the time of screening (Table 2). While RBC transfusions can be beneficial for anemic patients with MDS, they may increase the risk of developing iron overload. Although ferritin levels, the major iron storage protein, were increased in transfusion-dependent patients, T-scores were not associated with ferritin levels or transfusion dependency in patients with MDS (Table 3).

Finally, multiple regression analysis was performed to investigate whether T-scores, adjusted for calcium and phosphate levels, were influenced by age, sex, MDS duration, or transfusion dependency. The estimate remained significantly different for calcium and phosphate levels: however, these effects were independent of all other variables in the MDS cohort (Table 3).

### Association of MDS-associated mutations and IPSS-M score with BMD

The most frequent mutations in the MDS cohort were *ASXL1* (35%), *TET2* (34%), *RUNX1* (22%), *SF3B1* (21%), and *DNMT3A* (18%) (Supplementary Table S4). Mutation analysis is crucial for risk stratification according to IPSS-M, which also incorporates hematopoietic and cytogenetic features of MDS [15]. All patients were classified into low- or high-risk based on the IPSS-M, with high-risk patients having a higher probability of leukemic progression. Fifty-two patients were classified as low-risk and 43 patients as high-risk. Linear regression analysis showed that the IPSS-M score was not associated with T-scores (Table 3).

**Fig. 3:**
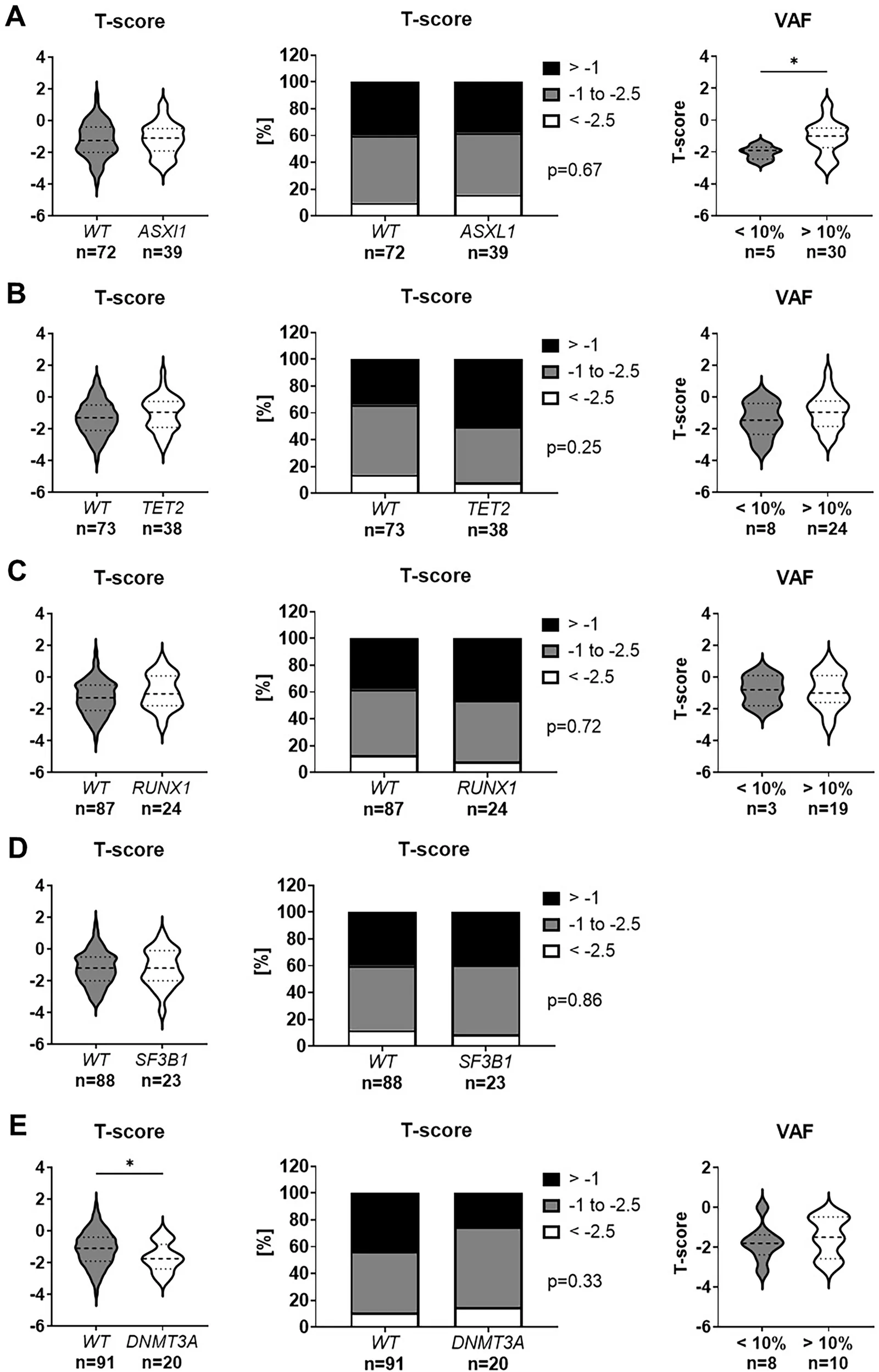
*DNMT3A* mutation is associated with a lower T-score in MDS. Calculated femoral neck T-scores of patients with MDS were depicted as raw data and classified into normal (>−1.0), osteopenia (between −1.0 and −2.5), and osteoporosis range (<−2.5) at baseline. The T-score distribution of **A** *ASXL1*, **B** *TET2*, **C** *RUNX1*, **D** *SF3B1*, or **E** *DNMT3A* mutation compared to patients with MDS with the wild-type (WT) allele was evaluated at baseline. In addition, the mutation was stratified by variant allele frequency (VAF, <10% and >10%) if the percentage was known from the mutation analysis. Since only one patient with MDS and *SF3B1* mutation had a VAF < 10%, no violin plot can be shown. Data of T-score classification are shown as percentage distribution. Statistical analysis was performed by Chi-squared test. All other data are shown as median with interquartile range in violin plots. The Mann–Whitney U test was used for the comparison of two groups. *p < 0.05.

To test whether specific genetic alterations were linked with lower T-scores, the MDS cohort we stratified into patients without detectable mutations and those harboring mutations in *ASXL1* (*n* = 39), *TET2* (*n* = 38), *RUNX1* (*n* = 24), *SF3B1* (*n* = 23), and *DNMT3A* (*n* = 20). The presence of the above-mentioned mutations did not change the distribution of T-score proportion compared to patients without mutations. However, analysis of absolute T-scores showed that patients with a *DNMT3A* mutation had lower T-scores [−0.7; *p* < 0.05], while stratification by variant allele frequency (VAF, <10% or >10%), if the exact mutation burden was known, revealed no differences (Fig. 3A–E). Patients with a *DNMT3A* mutation exhibited similar RBC counts and comparable BSAP, calcium, and phosphate serum levels compared to patients without this mutation (Supplementary Fig. S3A–D). Interestingly, a VAF > 10% of *ASXL1* mutation was associated with higher T-scores [+0.9; *p* < 0.05] (Fig. 3A).

Mutation analysis of peripheral blood cells was available for 58 individuals of the control cohort. Of these, 33 controls had no detectable mutations, while CH of indeterminate potential (CHIP) occurred in 25 individuals. The presence of a mutation had no effect on the T-scores, RBC counts, or serum levels of BSAP, calcium, and phosphate. Stratification of the control cohort into individuals with and without the *DNMT3A* mutation showed no effect of the mutation on the T-scores (Supplementary Fig. S3E–J).

### Longitudinal changes of the T-score in patients with MDS

**Fig. 4:**
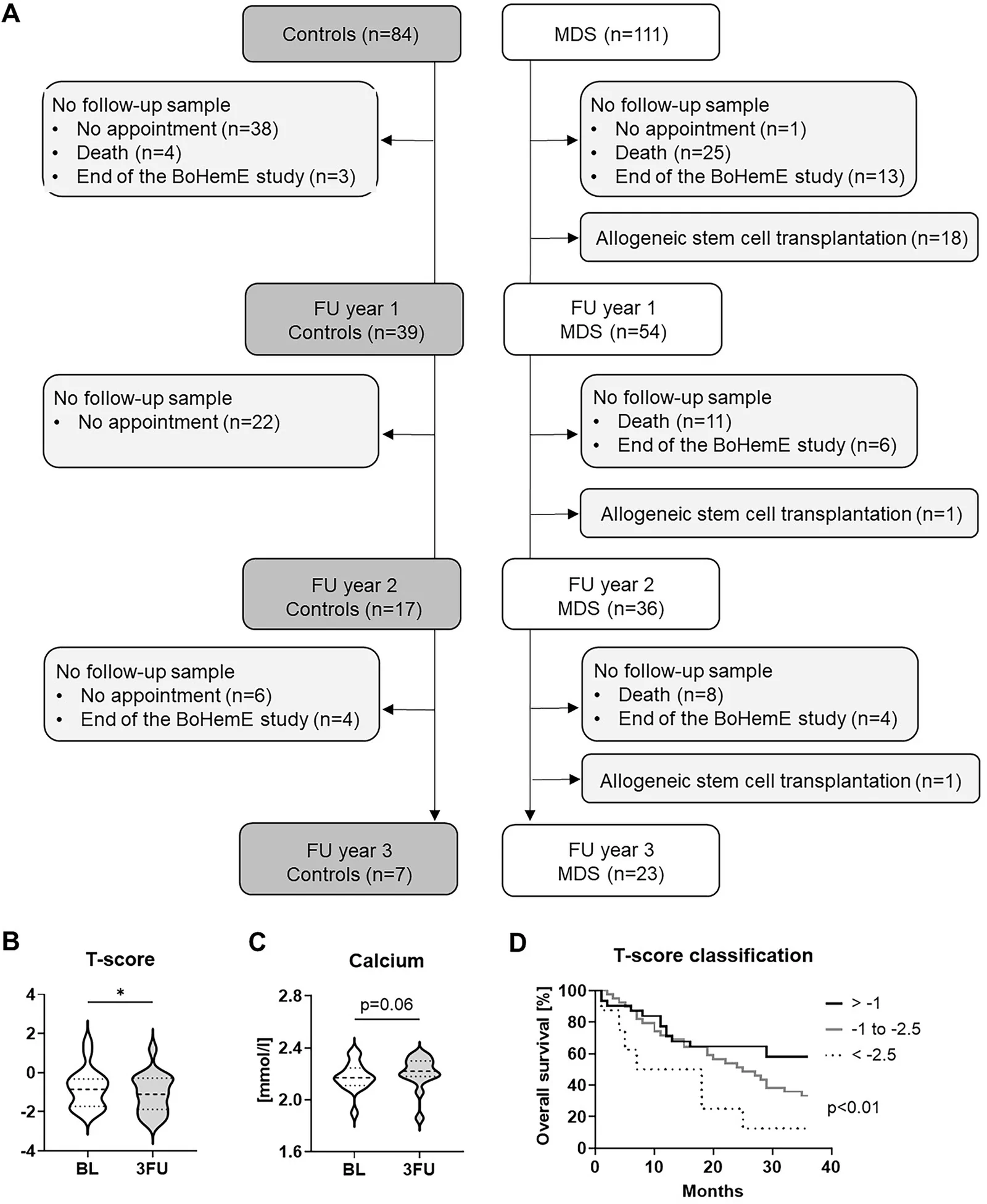
Osteoporotic T-score is associated with reduced overall survival in MDS. **A** Flowchart of the follow-up visits within the BoHemE study. Illustrated overview of the number of patients who presented for follow-up visits after 1, 2, and 3 years in the control and MDS cohort. **B** Calculated femoral neck T-scores and **C** albumin-adjusted calcium of patients with MDS from baseline (BL) were compared with 3-year follow-up (3FU) results. Data are shown as median with interquartile range in violin plots. The Mann–Whitney U test was used for the comparison of two groups. *p < 0.05. **D** Calculated femoral neck T-scores of patients with MDS were classified into normal (>−1.0), osteopenia (between −1.0 and −2.5), and osteoporosis (<−2.5) range at baseline and the overall survival was monitored over 3 years. Kaplan–Meier analyses were performed. Overall difference among the three groups was assessed using a log-rank tests for trend.

The 3-year follow-up (3FU) report comprised a small number of patients due to loss to follow-up, death, scheduled 3FU visits after study termination, or treatment with allogeneic stem cell transplantation (ASCT), which may positively affect bone metabolism [16]. Thus, 23 patients with MDS and 7 controls were included in the 3FU analysis (Fig. 4A). To avoid interference with bone-specific treatments, all patients who initiated specific osteoporosis therapy after study enrollment were excluded. In the control group, 5 of 7 individuals received bisphosphonates or denosumab, precluding meaningful analysis. Among patients with MDS, 2 of 23 received bisphosphonate therapy, and 3 additional patients did not undergo DXA scanning at 3FU. Thus, data from 18 patients with MDS were available for analysis (Supplementary Table S5). Compared to baseline, femoral neck T-scores decreased by −0.3, but RBC counts and serum levels of calcium, BSAP, and phosphate remained unchanged (Fig. 4B, C, Supplementary Fig. S4A–C). At baseline, none of these 18 patients had osteoporosis, 9 had osteopenia and 9 had normal T-scores. After 3 years, 1 patient was diagnosed with osteoporosis, 9 had osteopenia, and 8 had normal T-scores (Supplementary Table S5).

### Bone health and overall survival

Overall survival in the MDS cohort was analyzed according to T-score categories (normal, osteopenia, osteoporosis). Thirty-three patients from the baseline cohort (*n* = 111) were excluded from this analysis due to ASCT or because their projected 3FU occurred after study termination. Among the remaining patients with MDS included in survival analysis, 31 had normal T-scores, while 39 had osteopenia, and 8 had osteoporosis. Kaplan–Meier survival analysis demonstrated that normal T-scores were associated with improved overall survival in patients with MDS [*p* < 0.01]. After 3 years mortality rates were 42% in patients with normal T-scores, 67% in those with osteopenia, and 88% in those with osteoporosis (Fig. 4D).

### Association of QoL with bone health

To assess whether bone health affected the QoL, all study participants were asked to complete the EORTC QoL questionnaire once per year. At baseline, 79 questionnaires were evaluable from controls (94% reply rate) and 91 from patients with MDS (82% reply rate). As controls underwent hip replacement surgery for osteoarthritis associated with pain and impaired mobility, we used QoL data from the 1-year post-operative follow-up (*n* = 37). As expected, 6–12 months after surgery, the physical functioning [*p* < 0.0001] and role functioning [*p* < 0.001] improved, while pain scores decreased [*p* < 0.0001], resulting in higher overall QoL [*p* < 0.01] (Supplementary Table S6). Compared to controls, patients with MDS had lower emotional functioning [*p* < 0.01], and higher symptoms scores for fatigue [*p* < 0.01] and appetite loss [*p* < 0.05]. Overall, QoL was significantly reduced in patients with MDS [*p* < 0.01] (Table 4). However, QoL did not correlate with T-scores (Supplementary Table S7). During follow-up, social functioning decreased [*p* < 0.05] and pain scores increased [*p* < 0.01] at 3FU in the MDS cohort (*n* = 16) compared to baseline. Overall, QoL remained unchanged despite the lower T-score (Supplementary Table S6).

**Table 4:** Calculated scores of the EORTC QLQ-C30 in the control one year after the hip replacement and MDS cohort at baseline.

	Controls Median (IQR)	MDS cohort Median (IQR)	*p*-value
Functional scales^a^			
Physical	87 (70–93)	73 (53–93)	0.08
Role	83 (67–100)	67 (33–100)	0.35
Emotional	100 (71–100)	75 (57–92)	**<0.01**
Cognitive	100 (75–100)	83 (67–100)	0.23
Social	100 (67–100)	83 (50–100)	0.33
Symptom scales^b^			
Fatigue	17 (0–33)	33 (11–64)	**<0.01**
Nausea and vomiting	0 (0)	0 (0)	**<0.05**
Pain	33 (0–42)	33 (0–67)	0.80
Dyspnea	0 (0–33)	33 (0–67)	0.09
Insomnia	24 (35)	35 (36)	0.09
Appetite loss	0 (0)	33 (0–33)	**<0.05**
Constipation	0 (0)	0 (0–33)	0.10
Diarrhea	0 (0)	0 (0)	0.08
Financial difficulties	0 (0)	0 (0–17)	0.06
Global health status/Quality of life^a^	75 (59–83)	58 (42–75)	**<0.01**

Values in bold (<0.05 and <0.01) indicate significant changes.

^a^Score range from 0 to 100, a high score represents a better level of function or quality of life.

^b^Score range from 0 to 100, a high score represents a worse level of symptoms.

Finally, we assessed whether the QoL is associated with age, sex, MDS duration, IPSS-M, or transfusion dependency using linear regressions. Men with MDS had a higher QoL compared to women with MDS [β: 8.90; *p* < 0.05], whereas transfusion dependency was associated with a lower QoL [β: −13.50; *p* < 0.01]. In multiple regression analysis including T-scores, sex, and RBC transfusion dependency remained independently associated with QoL (Supplementary Table S7).

## Discussion

The BoHemE study highlights impaired bone health in patients with MDS, as reflected by lower T-scores, and identifies factors associated with or independent of this finding.

Given the close interactions within the osteo-hematopoietic niche, we hypothesized that CH and therapeutic interventions in patients with MDS may influence bone health. Our previous retrospective study demonstrated that patients with MDS were more frequently treated with osteoporosis medications compared to hematological healthy individuals [11]. However, it remained unclear whether age, sex, molecular mutations, risk score, transfusion dependency, or MDS duration affect bone health in MDS. To address these questions, the BoHemE study was initiated to investigate bone health and bone-specific markers in patients with and without MDS. In addition, patients with MDS were monitored over a 3-year follow-up period.

In our study, the distribution of sex was balanced in the control cohort, whereas significantly more men were included in the MDS cohort, reflecting the higher incidence of MDS in men compared to women [17]. In contrast, osteoporosis is more prevalent in women [9], primarily due to estrogen deficiency after menopause [18]. In the age-matched groups, women regardless of MDS status exhibited lower T-scores than men. The absence of osteoporosis cases in our control cohort in the femoral neck could be age-dependent, as osteoporosis is diagnosed in only 4% of men aged 70–79 years [9] while the median age of our male control cohort was 68 years. Alternatively, hip osteoarthritis may have masked osteoporotic T-scores at this site, although both sides were evaluated to minimize this potential bias. Notably, anemia is associated with osteoporosis during aging [19–21], and here we show for the first time, that patients with MDS exhibit lower T-scores.

Although CTX-I and osteocalcin levels did not differ between patients with MDS and controls, lower T-scores within the MDS cohort were associated with higher levels of these markers. This is partly consistent with other osteoporosis studies, in which osteocalcin is normal or slightly elevated, whereas CTX-I is typically increased because bone remodeling is disturbed, with bone resorption dominating [22, 23]. Thus, patients with MDS depict a distinct bone metabolism phenotype as reflected by serum bone turnover markers. In our study, albumin-adjusted serum calcium levels were reduced in both women and men with MDS compared to controls. However, these lower calcium levels were not attributable to increased creatinine levels or vitamin D deficiency, and did not trigger secondary hyperparathyroidism [24, 25]. A low calcium level appears to be advantageous in MDS, as regression models showed a negative correlation with the T-score. Furthermore, phosphate, although normal compared to controls, influenced the T-score along with calcium. While hypocalcemia and hyperphosphatemia occur in severe CKD [26, 27], individuals with an eGFR <45 ml/min were excluded from this study. Moreover, the decreased BSAP levels observed in patients with MDS, together with low calcium availability may point toward impaired bone matrix mineralization. Consistent with this, impaired bone mineralization leading to increased non-mineralized bone matrix has previously been reported in patients with MDS [28]. Taken together, these findings suggest that reduced bone formation may contribute to decreased BMD and impaired bone health in MDS, although histomorphometric analyses are required to confirm this hypothesis. Nevertheless, it is unclear why bone turnover markers remain unaltered in MDS despite the presence of bone loss. MDS is frequently preceded by a prolonged phase of clonal cytopenia of undetermined significance, which persists for years and may affect bone homeostasis. Compensatory mechanisms during this preclinical phase may mask changes of bone turnover markers. We observed a similar phenomenon in our MDS mouse model. Before the onset of MDS, biochemical markers of bone formation were markedly elevated, but declined following MDS onset, whereas biochemical markers of bone resorption remained unchanged despite a reduction in bone-resorbing cells [28]. A comparable mechanism may also exist in patients with MDS.

In addition, we assessed whether specific molecular alterations [29, 30] are associated with lower T-scores in controls. Despite previous evidence [31–34], the presence of CHIP in controls was not associated with differences in T-scores. This discrepancy may be explained by relatively younger median age of our control cohort (68 years). Furthermore, due to limited group sizes, stratifications by specific mutations were not feasible. In the MDS cohort, patients harboring *ASXL1* and *TET2* mutations did not exhibit lower T-scores. On the contrary, *ASXL1* mutations with a VAF (>10%) were associated with higher T-scores, whereas the presence of a *DNMT3A* mutation was associated with lower T-scores in MDS. As *DNMT3A* mutations have been associated with bone loss and increased osteoclast activity [31], future studies in larger cohorts should include detailed analyses of circulating osteoclast markers. In BoHemE study, the presence of CHIP was not associated with differences in T-scores, despite its established links to inflammation, autoimmune diseases, and cardiovascular disorders [8, 33, 35]. The differing effects of specific molecular mutations on the T-scores between MDS and controls may reflect differences in cell-cell interactions within the bone microenvironment, which vary between controls, controls with CHIP, and MDS [4, 36]. Finally, the presence of CH may have distinct effects on bone health in controls compared to patients with MDS.

In the follow-up periods, none of the patients with MDS experienced severe bone loss. However, mortality was highest among patients with osteoporosis at baseline. Nevertheless, there was no association between low T-scores and disease severity (IPSS-M score), whereas a high-risk score is known to be negatively associated with the overall survival [37]. Furthermore, overall QoL did not decline at 3FU. Although social functioning decreased and pain scores increased over the 3FU period, these changes may be attributable to the heterogenous treatment regimens in MDS. Surprisingly, QoL did not differ between T-score groups in patients with MDS, which contrast with a previous report demonstrating a negative correlation between T-scores and QoL [10].

Despite its major strengths, including the prospective design and the well-characterized cohorts, this study has limitations. As MDS is a severe disease with a high mortality rate [15], the number of patients at 3FU was limited. The small cohort size restricted sub-group analyses for falls and fractures, and these events were self-reported. Additionally, it would be interesting to determine whether the fall resulted in a fracture or whether the fracture occurred independently of a fall, e.g. with spinal fractures. The marked discrepancy between the controls and patients with MDS regarding falls and fractures could also be attributable to the higher risk of falling associated with gait instability in patients with osteoarthritis, and/or cautious mobility in patients with MDS because of the fatigue. Of note, DXA scans were performed in Dresden and Leipzig. At both sites, daily quality control phantom scans confirmed consistent and valid BMD measurement during the whole study period. However, cross-calibration phantom scans between the two sites to estimate and adjust for inter-machine differences were not performed. Nevertheless, given the observed association between MDS and impaired bone health, our findings suggest that bone health assessment should be included into the clinical management of MDS. BMD evaluation using DXA may facilitate early detection of osteoporosis. Increased awareness among hematologists regarding fracture risk, physical function, and QoL implications may enable timely preventive measures such as lifestyle counseling, supplementation strategies, or initiation of osteoporosis therapy. Future prospective studies are needed to define optimal screening and evidence-based management strategies for bone health in MDS.

Taken together, the key findings of this prospective BoHemE study include lower BMD in women with MDS and in patients with a *DNMT3A* mutations. The poor prognosis in patients with MDS and established osteoporosis warrant clinical attention and highlights the need for further optimization of their management.

## Supplementary information

Supplemental Material

## Data Availability

The described methods and generated data of the current study are available from the corresponding authors upon request.
