# Supplementary material for: Bone health in myelodysplastic neoplasms: results from the prospective longitudinal BoHemE study
Source: Leukemia. 2026 Oct 9;40(11):2573–83. doi: 10.1038/s41375-026-03125-8 (PMC13713548; doi:10.1038/s41375-026-03125-8)
Supplement: Supplementary file 1 [file 41375_2026_3125_MOESM1_ESM.pdf]

Supplementary information: Tables

Table S1. Inclusion and exclusion criteria for the BoHemE study.

<table>
<tr><th></th><th>Controls</th><th>MDS cohort</th></tr>
<tr><td rowspan="2">Inclusion Criteria</td><td colspan="2">Age ≥ 60 years</td></tr>
<tr><td>• Undergoing hip replacement therapy
• Normal blood count defined by hemoglobin (women: > 7.4 mmol/l; men: > 8.1 mmol/l) and platelet count > $100*10^9$/l</td><td>• Diagnosis of MDS (according to WHO)</td></tr>
<tr><td rowspan="2">Exclusion Criteria</td><td colspan="2">• Renal insufficiency with ab eGFR < 45 ml/min
• Liver cirrhosis Child-Pugh B or C
• Active infection (HIV, hepatitis B or C, tuberculosis)
• Heart insufficiency NYHA III or IV or severe cardiac valve disease
• Dementia defined as MMSE score of < 24</td></tr>
<tr><td>• Diagnosis of MDS or AML prior to study</td><td>• Other hematological diseases (AML, CLL, lymphoma)</td></tr>
</table>

WHO: World Health Organization; eGFR: estimated glomerular filtration rate; NYHA: New York Heart Association; MMSE: Mini-Mental State Examination; AML: acute myeloid leukemia; CLL: chronic lymphoid leukemia, CMML: chronic myelomonocytic leukemia.

Table S2. Characteristics of patients with MDS and CMML.

	MDS (n=96)	CMML (n=15)
**Sex, *n***		
**Women**	32 (33%)	8 (53%)
**Men**	64 (67%)	7 (47%)
**Age, years**		
**All**	69 (65-73)	68 (65-70)
**Women**	69 (63-74)	68 (67-71)
**Men**	69 (65-73)	65 (61-70)
**BMI, [kg/m^2]**		
**All**	26.6 (24.2-28.3)	26.0 (23.3-28.0)
**Women**	26.5 (23.8-28.2)	24.9 (21.2-27.0)
**Men**	26.8 (24.5-29.2)	28.0 (25.6-30.9)
**T-score**		
**All**	-1.3 (-2.1 to -0.5)	-0.6 (-1.9 to -0.1)
**Women**	-1.6 (-2.2 to -1.0)	-1.2 (-2.0 to -0.5)
**Men**	-1.1 (-1.9 to -0.4)	-0.4 (-1.0 to 0.0)
**Hematological parameters**		
**Red blood cells [10^{12}/l]**	2.85 (2.43-3.46)	3.40 (2.76-4.18)*
**Leukocytes [10^9/l]**	3.03 (2.04-4.65)	10.02 (4.10-28.59)****
**Platelets [10^9/l]**	132 (63-229)	115 (53-197)
**Transfusion-dependent, *n***		
**Yes**	32 (33%)	1 (7%)
**No**	64 (67%)	14 (93%)
**Genetic alterations, *n***		
***ASXL1* mutation**	31 (32%)	8 (53%)
***DNMT3A* mutation**	20 (21%)	0
***RUNX1* mutation**	19 (20%)	5 (33%)
***SF3B1* mutation**	23 (24%)	0
***TET2* mutation**	30 (31%)	8 (53%)

For continuous variables median (1st-3rd quartile) are given. *p<0.05; ****p<0.001.

Table S3. Association between the T-score and bone-specific parameters, age, or sex within the controls.

	Sample size, *n*	β	p-value
**Linear regression with T-score as the outcome**			
**CTX-I (log)**	79	-0.05	0.81
**Osteocalcin (log)**	82	-0.05	0.77
**BSAP (log)**	54	-0.05	0.74
**Calcium**	74	-1.30	0.31
**Phosphate**	79	-0.82	0.23
**PTH**	51	-0.01	0.09
**25-OH D3**	67	0	0.98
**Age**	84	-0.03	**<0.05**
**Age**			
**60 - 69 years**	48	ref.	
**≥ 70 years**	36	0.44	0.06
**Sex**			
**women**	46	ref.	
**men**	38	0.32	0.16

CTX-I, cross-linked C-telopeptide of type I collagen; BSAP, bone-specific alkaline phosphatase, PTH, parathyroid hormone, 25-OH D3, 25-hydroxyvitamin D3; log, before use values were converted to logarithms; ref, reference; β, estimate.

Table S4. Frequency of mutation in the MDS cohort.

Gene	WT allel, *n*	Mutation, *n*
ASXL1	72 (65%)	39 (35%)
TET2	73 (66%)	38 (34%)
RUNX1	87 (78%)	24 (22%)
SF3B1	88 (79%)	23 (21%)
DNMT3A	91 (82%)	20 (18%)

Table S5. Characteristics of patients with MDS at baseline and 3-year follow-up.

Study ID	Sex	Molecular genetics*		T-score, femoral neck
030-009	f	*TET2*	BL	1,6
			3FU	1,2
030-012	f	*TET2*	BL	-1,6
			3FU	-1,6
030-014	m	-	BL	-0,4
			3FU	-0,1
030-024	m	BL: *SF3B1*<br>1FU: *DNMT3A, SF3B1, TET2*	BL	-0,4
			3FU	-0,2
030-030	m	-	BL	-0,4
			3FU	-0,3
030-043	m	*SF3B1*	BL	-0,1
			3FU	-0,4
030-067	m	*SF3B1*	BL	-1,7
			3FU	-0,8
030-093	m	-	BL	-0,6
			3FU	-1,1
030-110	m	*SF3B1*	BL	0,0
			3FU	-0,2
030-139	f	-	BL	-1,8
			3FU	-2,1
030-151	f	*SF3B1*	BL	-1,2
			3FU	-1,2
030-155	m	*TET2*	BL	-0,9
			3FU	-1,8
030-165	m	-	BL	1,0
			3FU	-0,6
077-001	f	-	BL	-0,8
			3FU	-1,8
077-003	f	-	BL	-2,1
			3FU	-2,4
077-036	m	*TET2*	BL	-1,0
			3FU	-1,1
077-037	f	*DNMT3A*	BL	-1,8
			3FU	-2,3
077-040	f	*DNMT3A, RUNX1*	BL	-2,1
			3FU	-2,6

*Only mutation in ASXL1, DNMT3A, RUNX1, SF3B1, and TET2 were analyzed.
F, women; m, men; IPSS-M; BL, baseline; FU, follow-up.

Table S6. Calculated EORTC QLQ-C30 scores of participants of follow-up visits.

	Controls			MDS cohort		
	Baseline Median (IQR)	1-year FU Median (IQR)	p-value[1]	Baseline Median (IQR)	3-year FU Median (IQR)	p-value[1]
**Functional scales[#]**						
**Physical**	60 (47-67)	87 (70-93)	**<0.0001**	73 (47-87)	50 (33-82)	0.24
**Role**	50 (33-67)	83 (67-100)	**<0.001**	67 (17-100)	50 (17-100)	0.97
**Emotional**	75 (54-100)	100 (71-100)	0.07	79 (42-98)	71 (37-92)	0.35
**Cognitive**	100 (83-100)	100 (75-100)	0.57	83 (71-100)	83 (67-100)	0.86
**Social**	67 (33-83)	100 (67-100)	**<0.05**	83 (33-100)	42 (21-100)	**<0.05**
**Symptom scales[§]**						
**Fatigue**	33 (11-44)	17 (0-33)	0.08	33 (11-67)	56 (11-67)	0.96
**Nausea and vomiting**	0 (0)	0 (0)	0.74	0 (0)	0 (0-17)	0.25
**Pain**	67 (50-92)	33 (0-42)	**<0.0001**	33 (0-67)	50 (21-67)	**<0.01**
**Dyspnea**	0 (0-33)	0 (0-33)	0.43	33 (0-67)	33 (0-67)	1.00
**Insomnia**	33 (0-67)	0 (0-33)	**<0.05**	33 (0-67)	33 (33-67)	0.11
**Appetite loss**	0 (0)	0 (0)	0.49	0 (0-33)	0 (0-33)	0.66
**Constipation**	0 (0)	0 (0)	0.93	0 (0)	0 (0-67)	0.63
**Diarrhea**	0 (0)	0 (0)	0.54	0 (0)	0 (0-33)	0.06
**Financial difficulties**	0 (0)	0 (0)	0.62	0 (0)	0 (0-25)	1.00
**Global health status/Quality of life[#]**	50 (33-67)	75 (59-83)	**<0.01**	50 (44-79)	63 (50-83)	0.64

[#]Score range from 0 to 100, a high score represents a better level of function or quality of life.

[§]Score range from 0 to 100, a high score represents a worse level of symptoms.

[1]Baseline vs. FU of the same cohort

Table S7. Association between the quality of life and T-score, age, sex, or MDS characteristics within the MDS cohort.

	**Sample size, *n***	**r**	**p-value**
**Correlation with QoL**			
**T-score**	91	0.03	0.78
	**Sample size, *n***	**β**	**p-value**
**Linear regression with QoL as the outcome**			
**Age**			
**60 - 69 years**	51	ref.	
**≥ 70 years**	40	-5.90	0.18
**Sex**			
**women**	36	ref.	
**men**	55	8.90	**<0.05**
**Time interval since MDS diagnosis (log)**	90	-0.60	0.69
**IPSS-M**			
**low risk**	42	ref.	
**high risk**	37	-4.50	0.33
**Transfusion dependency**			
**no**	63	ref.	
**yes**	28	-13.50	**<0.01**
**Multiple linear regression with QoL as the outcome***			
**Sex**			
**women**	36	ref.	
**men**	55	10.50	**<0.05**
**Transfusion dependency**			
**no**	63	ref.	
**yes**	28	-14.60	**<0.01**
**T-score**	91	-0.50	0.79

QoL, quality of life; r = Spearman correlation coefficient; log, before use values were converted to logarithms; ref, reference; β, estimate.
*R^2 = 0.15 for this model

Supplementary information: Figures

Figure S1: Blood count of controls, patients with MDS or CMML. The number of (A) red blood cells, (B) platelets, and (C) leucocytes were analyzed by Sysmex (Sysmex, Norderstedt, Germany). Data are shown as median with interquartile range in violin plots. Mann-Whitney U test was used for statistical analysis. ****p<0.0001.

Figure S2: Comparison of the T-score distribution and bone parameters of patients with MDS and CMML. DXA scans were performed in all participants at baseline. Bone mineral density was transformed into T-scores, and subjects were classified as normal (T-score > −1.0), osteopenia (T-score between −1.0 and −2.5), and osteoporosis (T-score < −2.5) according to WHO classification. (A) Comparison of the femoral neck T-score distribution in patients with MDS and CMML. (B) Cross linked C-telopeptide of type I collagen (CTX-I) and (C) osteocalcin were determined in plasma samples. (D) Bone-specific alkaline phosphatase (BSAP), (E) albumin-adjusted calcium, and (F) phosphate were measured in serum using ELISA. (B) Data are shown as percentage distribution. Statistical analysis was performed by Chi-squared test. All other data are shown as median with interquartile range in violin plots. The Mann-Whitney U test was used for the comparison of two groups.

Figure S3: Bone health is not affected by clonal hematopoiesis in controls. The MDS cohort was stratified regarding the presence of *DNMT3A* mutation and patients with the wild-type (WT) allele and (A) red blood cell count, (B) BSAP, (C) albumin-adjusted calcium, and (D) phosphate serum levels were compared between both groups. The control group was stratified regarding the presence of clonal hematopoiesis of indeterminate potential (CHIP) and no mutation (non-CHIP). (E) The femoral neck T-score, (F) red blood cell count, (G) BSAP, (H) albumin-adjusted calcium, and (I) phosphate serum levels were compared between clonal hematopoiesis of indeterminate potential (CHIP) and non-CHIP. The control cohort was stratified regarding the presence of *DNMT3A* mutation and controls with the WT allele. The femoral neck T-score was compared between the WT and *DNMT3A* group. Data are shown

as median with interquartile range in violin plot. The Mann-Whitney U test was used for the comparison of two groups.

Figure S4: Red blood cell counts and serum parameter stayed similar in the MDS cohort over 3 years. (A) Red blood cell count, (B) BSAP serum levels, and (C) phosphate serum levels of patients with MDS from baseline (BL) were compared with 3-year follow-up (3FU) results. Data are shown as median with interquartile range in violin plots. The Mann-Whitney U test was used for the comparison of two groups.

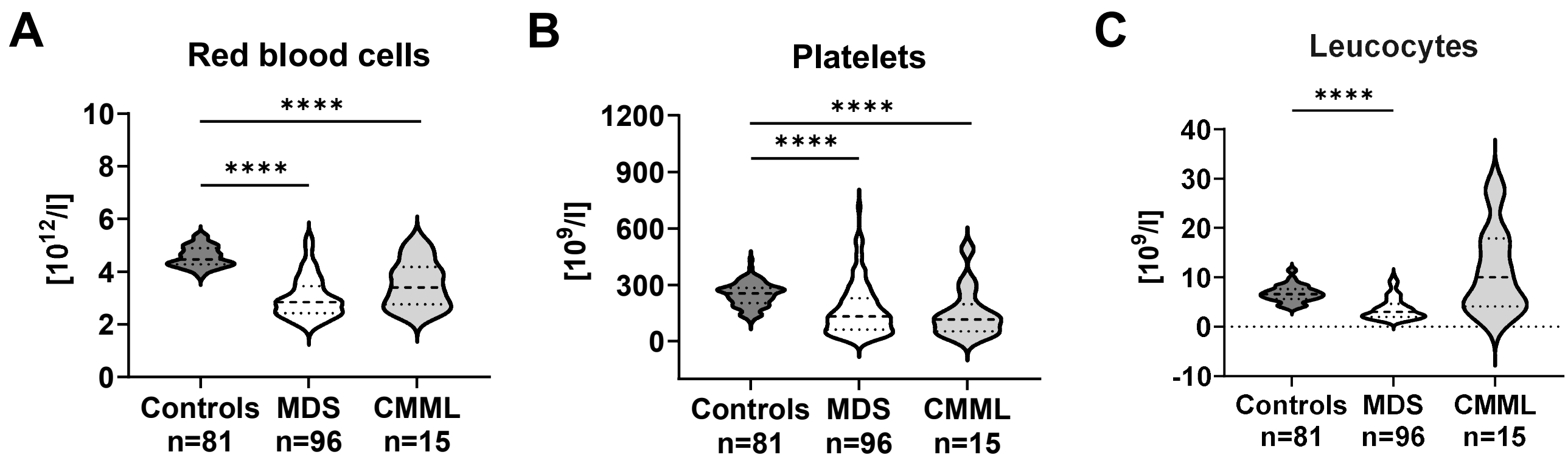

Supplementary Fig. 1.

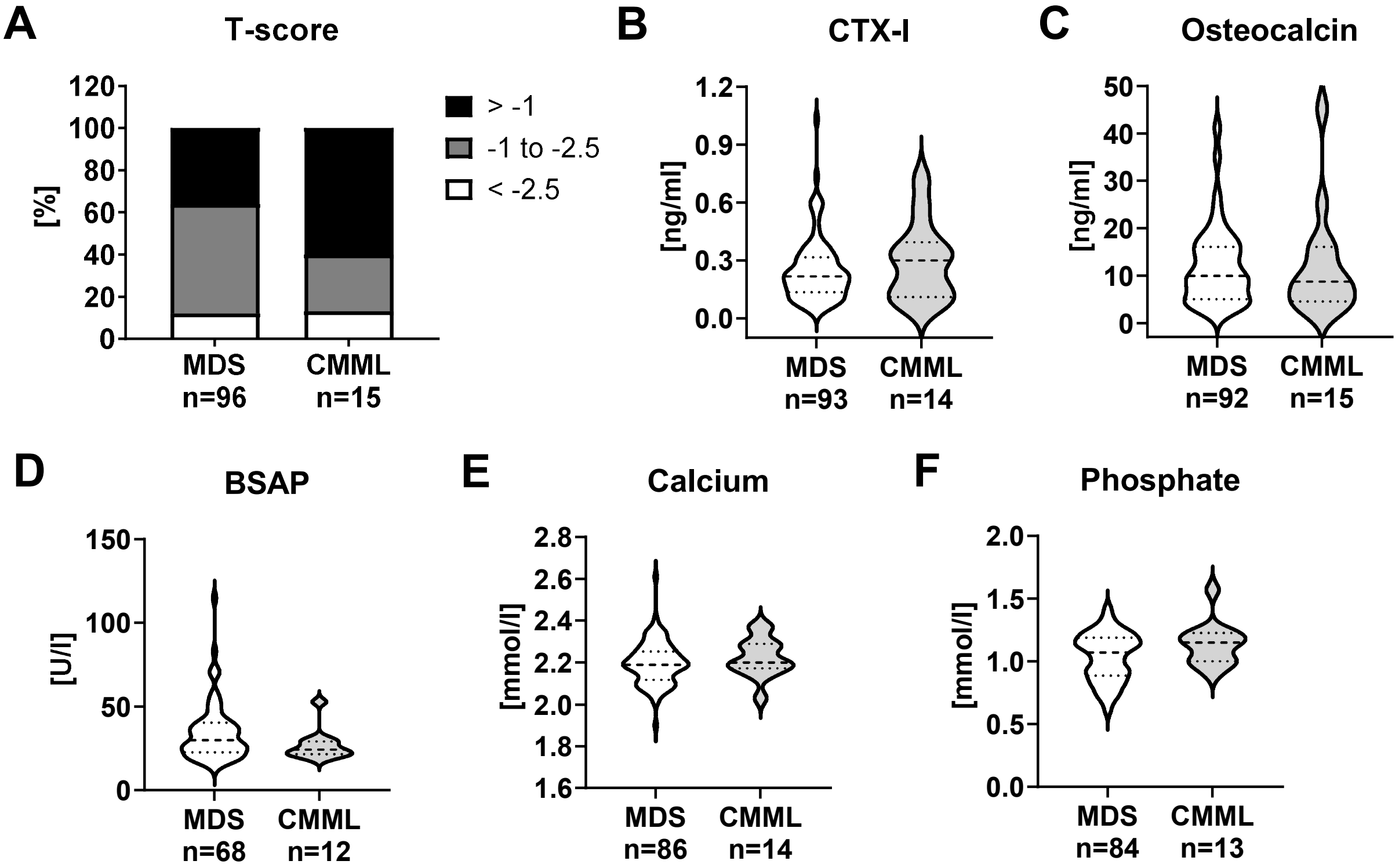

Supplementary Fig. 2.

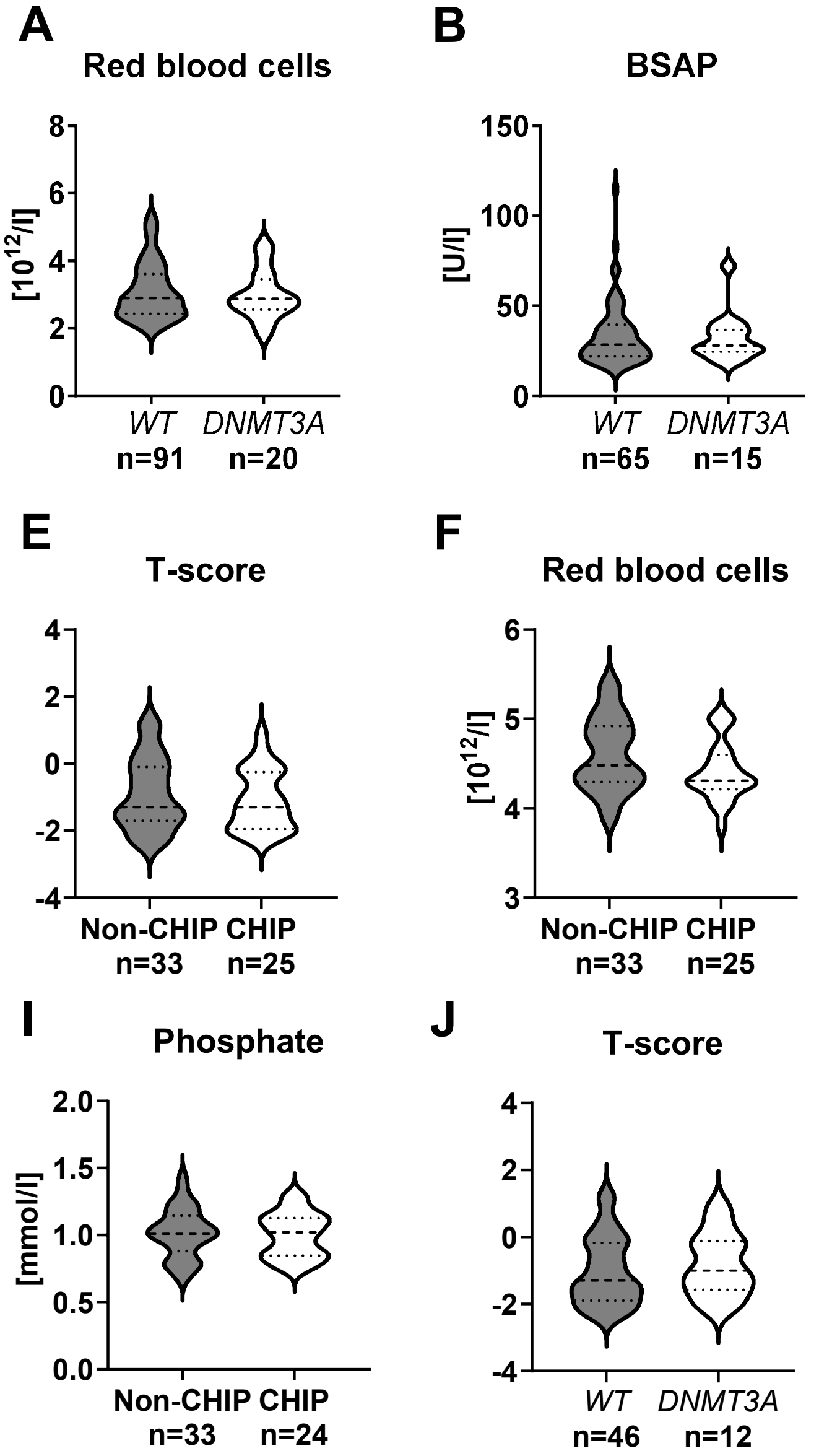

Supplementary Fig. 3.

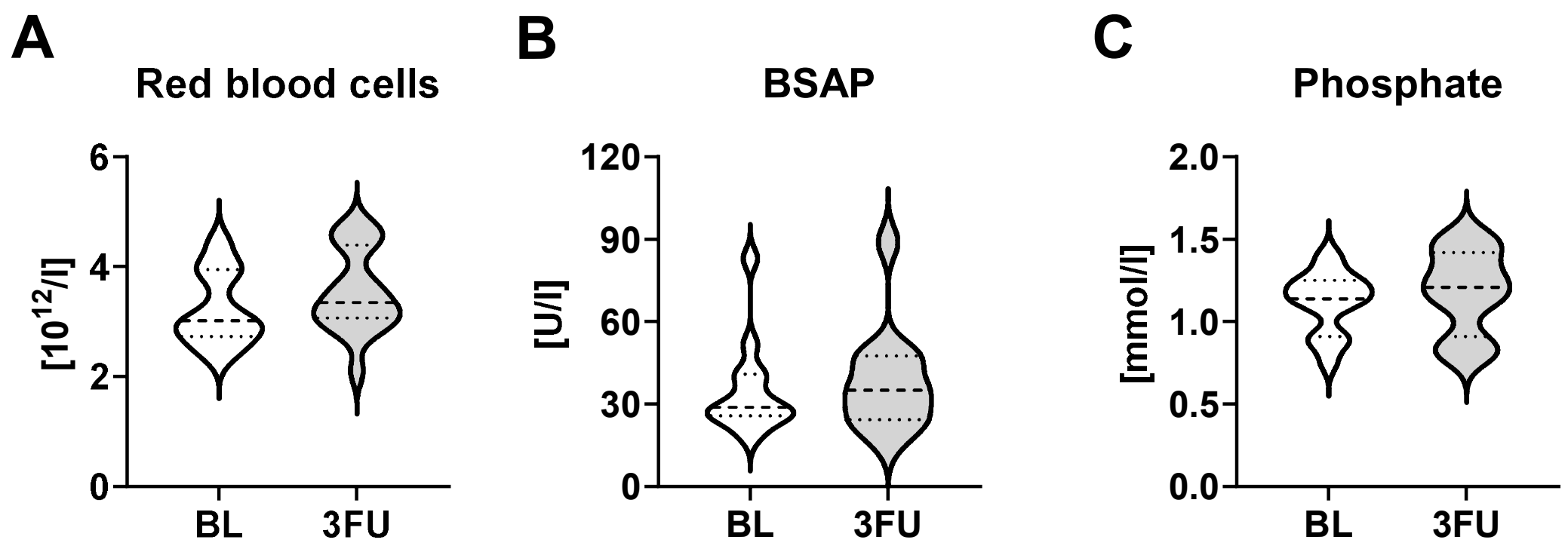

Supplementary Fig. 4.
